# The epidemiology of interstitial lung disease and its association with lung cancer

**DOI:** 10.1038/sj.bjc.6602061

**Published:** 2004-08-31

**Authors:** G Raghu, F Nyberg, G Morgan

**Affiliations:** 1 Department of Medicine, University of Washington Medical Center, Campus Box 356522, Seattle, WA 98195-6522, USA; 2 AstraZeneca R&D, Mölndal, Sweden; 3 Karolinska Institute, Stockholm, Sweden; 4 Royal North Shore Hospital, Sydney, Australia

**Keywords:** interstitial lung disease, epidemiology of ILD, lung cancer, non-small-cell lung cancer

## Abstract

The criteria and terminology for diagnosing interstitial lung disease (ILD), a diverse range of pulmonary fibrotic disorders that affect the alveoli of the lungs, have been variable and confusing; however, there have been recent major improvements to an internationally agreed classification. Evidence from recent analyses of populations suggests that the incidence and prevalence rates of ILD are on the increase, particularly when the broad definition of ILD is used. In most patients with ILD a cause is not identified; nevertheless, among the established causes are a number of drug therapies and infections. Occupational causes are lessening in importance, while cigarette smoking is now an established risk factor. Radiation therapy for cancer is a well-established cause of ILD that usually, but not always, localises within the radiation portal and may occur later after completion of therapy. Similarly, exposure to drugs long after radiation therapy may be an aetiological factor for the development of ILD later in life, although the magnitude of this risk requires further epidemiological investigation. The possibility that ILD and lung cancer are associated has been recognised for >50 years, but it remains unclear whether ILD precedes lung cancer or vice versa. In this review, we examine the epidemiology of ILD and the basis for its association with lung cancer.

## INTRODUCTION

Interstitial lung disease (ILD) refers to a diverse range of pulmonary fibrotic disorders that affect the alveoli of the lungs. Approximately two-thirds do not have a known cause (idiopathic), while one-third result from known endogenous or exogenous causes, including environmental/occupational factors, infections, drugs and radiation. Variation in the classification of ILDs, both historically and internationally, has not aided diagnosis, but recent consensus guidelines to both diagnosis and classification, together with a new nomenclature, offer an opportunity for greater precision. In the light of this new classification, this review examines the epidemiology of ILD and the evidence for its potential increase in prevalence.

## CLASSIFICATION AND DIAGNOSIS OF ILD

A considerable advance has occurred in the recent consensus document on the definition of ILD, which is also known as diffuse parenchymal lung disease. Interstitial lung disease encompasses a range of disorders, the most common of which is idiopathic interstitial pneumonia (IIP) (American Thoracic Society, 2002). Idiopathic interstitial pneumonia is a distinct disease that can be classified into seven types (Table 1

**Table 1:** Classification of IIPs (American Thoracic Society, 2002)

**IPF/CFA**	**IIP other than IPF**
	Desquamative interstitial pneumonia
	Acute interstitial pneumonia
	Nonspecific interstitial pneumonia
	Respiratory bronchiolitis interstitial lung disease
	Cryptogenic organising pneumonia
	Lymphocytic interstitial pneumonia

) (American Thoracic Society, 2002).

The new classification has been devised to overcome the lack of an international standard that has resulted in variable and confusing diagnostic criteria and nomenclature. Internationally, there had been many different terms for ILD; for example, in Japan the term interstitial pneumonia (IP) was used, while cryptogenic fibrosing alveolitis (CFA) was in use in the UK but idiopathic pulmonary fibrosis (IPF) was preferred in the USA. The preferred old terms were IPF and CFA, with IP often used interchangeably (Katzenstein and Myers, 1998; Poletti and Kitaichi, 2000). Nonstandard disease classifications have created difficulties in determining the rates of prevalence and incidence of ILD. Internationally, as coding for the disease has varied, it has proved quite difficult to apply the new classification to the many population registers.

Despite the guidelines for diagnosis with clearly described criteria, ILD remains a diagnosis of exclusion requiring extensive investigation, which is seldom possible. Many investigations are required to differentiate ILD from other diseases (Chernecky and Sarna, 2000). Infections can be ruled out through blood cultures, sputum and urine analyses, echocardiography can assess the likelihood of cardiac oedema and bronchoscopy, while bronchoalveolar lavage can reveal pulmonary haemorrhage. High-resolution computed tomography (HRCT) and pulmonary function tests are the main tests used to indicate parenchymal lung disease. However, the definitive investigation is a lung biopsy with detailed histopathology (Katzenstein and Myers, 1998). In clinical practice, only a small percentage of patients undergo lung biopsy, reflecting concerns about the complication of the procedure that includes prolonged pneumothorax (Hunninghake *et al*, 2001) and the fact that many patients are old and infirm.

When ILD is suspected, with use of clinical criteria and CT the sensitivity of the procedure is 72–77%, while specificity is higher at 72–84% as a result of the ability to exclude other diseases by these investigations (Hunninghake *et al*, 2001). While we have no information on the use of these investigations when a patient has ILD as a comorbidity of another disease, such as lung cancer, it is likely to be of similar efficacy.

### Incidence and prevalence rates for ILD

It is not possible to precisely estimate the incidence and prevalence rates of ILD for the previously given reasons. In addition, much of the available data are taken from registries or hospital clinics and therefore suffer from selection biases, making them unrepresentative of the general population (Mapel *et al*, 1998).

The most important registry study was undertaken in Bernalillo County, New Mexico, USA, using data from a dedicated ILD registry and employing broad case-identification procedures and systematic chart review (Coultas *et al*, 1994). The estimated incidence of ILD was 30 per 100 000 per year, with approximately one-third in the IPF category; the estimated incidence was slightly higher for men than women (Table 2

**Table 2:** Prevalence in 1988 and incidence in 1988–1990 of ILD in New Mexico (Coultas *et al*, 1994)

	**Prevalence per 100 000 (*n*)**		**Incidence per 100 000 per year (*n*)**	
**ILD category**	**Men**	**Women**	**Men**	**Women**
Occupational/environmental	20.8 (35)	0.6 (1)	6.2 (21)	0.8 (3)
Drug/radiation	1.2 (2)	2.2 (4)	1.8 (6)	1.1 (4)
Pulmonary haemorrhage syndromes	0.6 (1)	2.2 (4)	1.5 (5)	0.8 (3)
Connective tissue disease	7.1 (12)	11.6 (21)	2.1 (7)	3.0 (11)
*Pulmonary fibrosis*				
Fibrosis (post-inflammatory)	10.1 (17)	14.3 (26)	3.9 (13)	4.1 (15)
IPF	20.2 (34)	13.2 (24)	10.7 (36)	7.4 (27)
Interstitial pneumonitis	1.8 (3)	2.8 (5)	1.8 (6)	1.4 (6)
Sarcoidosis	8.3 (14)	8.8 (16)	0.9 (3)	3.6 (13)
Other	10.7 (18)	11.6 (21)	2.7 (9)	3.9 (14)
Total	80.9 (136)	67.2 (122)	31.5 (106)	26.1 (96)

*n*=number of cases in registry. Reprinted with permission from Coultas DB *et al* (1994). Copyright © 1994 American Lung Association.

) (Coultas *et al*, 1994). The estimated prevalence of ILD was almost three times as high as the incidence, which suggests a mean survival of approximately 3 years.

Very little data from population-based registries are available from other countries. The few published reports suggest international differences in the prevalence of IPF. It is estimated to be 4.1 per 100 000 in the Japanese Hokkaido registry (Munakata *et al*, 1994), 7–12 per 100 000 in the Czech Republic (Kolek, 1994) and 16–18 per 100 000 in the Finnish registry (Hodgson *et al*, 2002), compared with 20.2 per 100 000 in men and 13.2 per 100 000 in women in Bernalillo County (Coultas *et al*, 1994). It is unclear whether these variations reflect the disease prevalence or differences in registry methodology or completeness of case ascertainment.

Comparing the Bernalillo County data with earlier US estimates suggests an increase in the prevalence of IPF from 3–5 per 100 000 in 1984 (Crystal *et al*, 1984) to 20.2 and 13.2 per 100 000 in men and women, respectively, in 1994 (Coultas *et al*, 1994). This might reflect a true increase in IPF prevalence over 10 years, but it is also likely that earlier studies underestimated IPF prevalence because they were based on selected populations and because there was less awareness of ILD (Coultas *et al*, 1994). The advent of HRCT may have contributed to this increase in rate.

Mortality data have also been reported to show increasing trends in CFA and IPF mortality in England, Wales, Scotland, Australia and Canada, stable mortality rates in New Zealand and Germany, and a decrease in CFA mortality in the USA (Hubbard *et al*, 1996), although the latter may be due to coding practices as mortality from pulmonary fibrosis overall in the USA increased during the same period (Mannino *et al*, 1996). Even though these trends were not apparently due to changes in diagnostic criteria, mortality data are biased as they are based on death certificates that under-report the incidence and prevalence of ILD (Coultas and Hughes, 1996).

In line with these observed increases in the prevalence of ILDs, a recent study of the healthcare cost has shown a rise in rates. This retrospective cohort study of IPF was conducted using the integrated database of medical and pharmacy claims from private health plans in the USA from January 1996 to December 2000 (Weycker *et al*, 2002). Analyses were conducted using both a broad case definition (a medical claim with a diagnosis of IPF and no subsequent medical claim with diagnoses of other ILDs) and a narrow case definition (the same as the broad case definition, but with a medical claim for lung biopsy or CT of the thorax). Prevalence of IPF was determined from the existing number of health plan members with IPF in 2000, and incidence of IPF was determined from the number of newly diagnosed cases in 2000 and related to population size. Healthcare use and charges were estimated using all paid medical fees and pharmacy claims for 2000 for these IPF cases.

In keeping with the population-based reports above and the registries, the prevalence of IPF was estimated at 54 per 100 000 adults using the broad case definition and 17 per 100 000 adults using the narrow case definition. Idiopathic pulmonary fibrosis prevalence increased with age, with most patients aged ⩾65 years. Idiopathic pulmonary fibrosis incidence for 2000 was estimated to be 25 per 100 000 per year using the broad case definition and 10 per 100 000 per year using the narrow case definition. From what is known of clinical practice, the narrow definition is likely to be a lower limit estimate of the true occurrence of disease. The broad definition will probably include some false positives but will also have <100% specificity, so it may well be closer to the true incidence and prevalence. Overall, these data are consistent with the estimates from New Mexico (Coultas *et al*, 1994).

The mean patient healthcare charges related to IPF in 2000 were estimated at US$33 304 and US$40 707 per patient, based on the broad and narrow case definitions, respectively. Hospital admissions accounted for the main part of costs (71–73%), with outpatient charges accounting for only 20–22% of costs.

### Relative frequencies of parenchymal lung diseases comprising ILD

Several prospective European ILD registries have been established (Roelandt *et al*, 1995; Schweisfurth, 1996; Rizzato and Bariffi, 1999; Agostini *et al*, 2001; Thomeer *et al*, 2001a). Although these selective registries do not have complete case ascertainment in a defined population and thus are likely to underestimate the prevalence or incidence of ILD, they may allow useful comparisons of the relative frequency of the subcategories of diseases that make up ILD. Comparison of registries in Belgium, Italy and Germany, and in Bernalillo County highlighted similarities and differences (Table 3

**Table 3:** Comparison of subcategories of ILD across ILD/fibrosis registries (Thomeer *et al*, 2001b)

	**Proportion of all ILD cases in registry, %**					
	**Prevalent cases**			**Incident cases**		
**ILD category**	**New Mexico**	**Belgium**	**Italy**	**New Mexico**	**Belgium**	**Germany**
*Total ILD cases, n*	258	362	1138	202	264	234
Occupational/environmental						
Coal workers pneumoconiosis	3	NR	NR	0	NR	0.4
Other pneumoconiosis (asbestosis, silicosis, cobalt, etc.)	11	6	NR	10	7	2
Hypersensitivity pneumonitis	0	13	4	1	12	11
Drug	2	3	2^a^	3	5	3
Radiation	0.4	NR		1	NR	NR
Pulmonary haemorrhage syndromes (Goodpasture syndrome, Wegener granulomatosis and Churg–Strauss syndrome)	1	1	2	3	2	1
Connective tissue disease	13	7	NR	9	7	2
Sarcoidosis	12	31	30	8	26	35
IPF	22	17	37	31	19	32
Pulmonary fibrosis						
Fibrosis (post-inflammatory), interstitial pneumonitis	28	NR	NR	20	NR	NR
ILD, not otherwise defined	3	9	NR	10	10	5
Other (including BOOP, (C)EP, eosinophilic granuloma, etc.)	5	12	25	2	13	8

NR=not recorded (category not collected or not used); BOOP=bronchiolitis obliterans organising pneumonia; (C)EP=chronic eosinophilic pneumonia. Adapted with permission from Thomeer MJ *et al* (2001b).Copyright © 2001 European Respiratory Society Journals Ltd.

a Drug plus radiation therapy.

) (Thomeer *et al*, 2001b).

While IPF was a common subcategory of ILD in all registries, sarcoidosis made up a larger proportion of reported ILD cases in the European registries than the US registry (Demedts *et al*, 2001; Thomeer *et al*, 2001b). ILDs related to occupational and environmental exposures constituted a smaller proportion of registered ILD cases in the Italian registry than the other registries.

### Environmental factors that contribute to ILD

It is necessary to seek an explanation for these apparent international differences in the prevalence rates of the various forms of ILD and the potentially rising world rate. There are a number of important risk factors for ILD, cigarette smoking being the most common exposure associated with increased risk (Iwai *et al*, 1994; Baumgartner *et al*, 1997; Britton and Hubbard, 2000; Nagai *et al*, 2000). This is in contrast to occupational exposure, which is diminishing as a risk factor as a result of improved working environments. The most commonly recognised risk factors are exposure to dust from metal, wood, vegetables and animals, although exposures related to hairdressing, raising birds, and stone cutting and polishing have also been associated with ILDs (Iwai *et al*, 1994; Hubbard *et al*, 1996, 2000a; Baumgartner *et al*, 2000).

Pulmonary fibrosis and ILD can occur as a complication of certain drug exposures, including antidepressants, antiarrythmics (amiodarone), beta blockers, antibiotics, chemotherapeutic agents (e.g. bleomycin, mitomycin, cyclophosphamide and methotrexate), anticonvulsants and nonsteroidal anti-inflammatory drugs (Hubbard *et al*, 1998, 2000b; Foucher *et al*, 2003). There is even evidence that remote exposure to nonsteroidal anti-inflammatory drugs may be an aetiological factor that leads to pulmonary fibrosis later in life. However, clear and increased risks have been demonstrated with only a few drugs. This illustrates the value of careful clinical history taking. It also clearly shows the need for epidemiological work for agents such as those used for chemotherapy.

An association between CFA and Epstein–Barr virus has also been suggested (Vergnon *et al*, 1984). Indeed, there are data to associate other viral infections with ILD; for example, in Japan hepatitis C infection is a common association (Ueda *et al*, 1992).

It can be concluded that there is no single explanation for the apparent increased prevalence of ILD and for the differences between nations.

## ILD AS A COMORBIDITY OF LUNG CANCER

The association between ILD and lung cancer has been recognised since 1952 (Callahan *et al*, 1952). Whether ILD precedes lung cancer or vice versa, or if both sequences are common, remains unclear.

### Lung cancer in ILD patients

Idiopathic pulmonary fibrosis patients have a higher incidence of lung cancer than the general population, with relative risks of 7.3 and 14.1 being reported in UK follow-up studies and a similar ratio of 5.3 for prevalence of lung cancer at death in a small Japanese autopsy study (Turner-Warwick *et al*, 1980; Matsushita *et al*, 1995; Hubbard *et al*, 2000c) (Table 4

**Table 4:** Lung cancer occurrence in ILD and non-ILD populations

**Study**	**Country**	**Population**	***n*^a^**	**LC occurrence (%)**	**Measure**	**Risk ratio of ILD *vs* non-ILD group or population**
Turner-Warwick *et al* (1980)	UK	CFA hospital	205	9.8	CI 5–10 years	
		General population	NA			RR 14.1^c^
Hubbard *et al* (2000c)	UK	CFA GPRD	890	4.4	CI 5 years	
		Control GPRD	5884	0.9		RR 7.31^d^
Munakata *et al* (1994)^b^	Japan	IIP registry	67	14.9	CI 3.75 years	
		COPD registry	108	1.9		RR 7.8^c^
Asada *et al* (1992)	Japan	IIP hospital patient series	212	6.1	Prevalence at diagnosis	
Takeuchi *et al* (1996)	Japan	IIP patient series	102	16.7	Prevalence at diagnosis	
			85	7.1	CI 5–10 years	
Ogura *et al* (1997)	Japan	IIP hospital patients	599	14.9	Prevalence at diagnosis	
			510	5.7	CI 4 years	
Stack *et al* (1972)	UK	CFA hospital patients	96	5.2	CI 4 years	
Wells and Mannino (1996)	USA	Pulmonary fibrosis death certificates		4.81	Frequency reported (as underlying or contributing cause of death)	
		General population death certificates		6.45		0.74
		COPD death certificates		10.1		0.48
		Asbestosis death certificates		26.6		0.18
Harris *et al* (1998)	UK	Pulmonary fibrosis death certificates		6		
		Pneumoconiosis death certificates		7		0.86
		Silicosis death certificates		8		0.75
		Asbestosis death certificates		43		0.14
Matsushita *et al* (1995)	Japan	UIP autopsies	83	48.2	Prevalence at autopsy	
		General autopsies	NA	9.1		5.3
Hironaka and Fukayama (1999)	Japan	IPF/UIP autopsy population	70	46	Prevalence at autopsy	

LC=lung cancer; CI=cumulative incidence; GPRD=general practice research database; UIP=usual interstitial pneumonia; NA=not available.

a Size of study population.

b Munakata *et al* report an extended follow-up of the population in Ohtsuka *et al* (1991), where the reported rates after shorter follow-up were slightly higher.

c RR=relative risk.

d RR=gender- and age-adjusted rate ratio.

). The prevalence of lung cancer at the time of IIP diagnosis has been reported in three Japanese patient series studies as 6, 17 and 15% (Asada *et al*, 1992; Takeuchi *et al*, 1996; Ogura *et al*, 1997).

Among those IIP patients initially free of lung cancer, the approximate cumulative lung cancer incidence over 3–10 years of follow-up ranged from 6 to 15% in Japanese IIP patient series and from 4 to 10% in the UK CFA patient series (Turner-Warwick *et al*, 1980; Ohtsuka *et al*, 1991; Munakata *et al*, 1994; Takeuchi *et al*, 1996; Ogura *et al*, 1997; Hubbard *et al*, 2000c).

Many Japanese studies on more or less selected populations of IIP patients reported a frequency of lung cancer that was mostly a combination of prevalence and incidence, making them difficult to interpret. They include two nationwide surveys (8.7 and 19.1%) and a large number of studies up to 1990 with a range from 8 to 65% and an overall frequency of 27% (reviewed in Takiguchi *et al*, 1993), as well as some later studies reporting similar frequencies in the approximate range 20–30% (Nagai *et al*, 1992; Takeuchi *et al*, 1996; Ogura *et al*, 1997).

Idiopathic pulmonary fibrosis patients also have a higher incidence of lung cancer than patients with chronic obstructive pulmonary disease (COPD), even though COPD is itself associated with lung cancer (Samet, 2000). An unadjusted risk ratio of 7.8 for lung cancer in IPF patients compared with COPD patients was reported from the Japanese Hokkaido registry data (Ohtsuka *et al*, 1991; Munakata *et al*, 1994).

However, two large death-certificate studies from the UK and the USA did not confirm the association between IPF and lung cancer risk. These studies found a lower report rate of lung cancer on the death certificates of individuals with pulmonary fibrosis mentioned as cause of death on the certificate, than for the general population or for patients with COPD mentioned (Wells and Mannino, 1996; Harris *et al*, 1998). However, death-certificate studies are problematic because only underlying conditions or those contributing to the death are coded. Negative bias arises because lung cancer is more likely to be included on the death certificate than IPF (Samet, 2000). Data from the Bernalillo County registry showed that ILD was mentioned on the death certificates of only 46% of ILD patients and concordance of diagnosis in those patients was 76% (Coultas and Hughes, 1996).

### An explanation of the association between ILD and lung cancer

Initially, studies focused on the hypothesis that IPF, as a result of scarring or other proliferative mechanisms, could lead to the secondary development of lung cancer (Samet, 2000; Ma *et al*, 2001).

Many occupational and environmental exposures have been associated with an increased risk of both ILD and lung cancer and have been investigated as common mechanisms to explain the association (Samet, 2000). Many studies report that patients with both IPF and lung cancer are generally heavy smokers (Mizushima and Kobayashi, 1995; Takeuchi *et al*, 1996; Aubry *et al*, 2002). However, smoking does not appear to fully explain the association between CFA and lung cancer. Hubbard *et al* (2000c) found that the lung cancer rate ratio of 7.3 (95% confidence interval 4.5–11.9) for CFA patients compared with population controls was not significantly changed by adjusting for smoking (relative risk 8.3) or by considering current smokers only (rate ratio 7.4).

As noted, an important hypothesis is that the diffuse inflammatory process of IPF may increase lung cancer risk (Samet, 2000). Cryptogenic fibrosing alveolitis produces chronic inflammation resulting in the remodelling of the lung and malignancy may be secondary to these chronic inflammatory and fibrotic processes (Matsushita *et al*, 1995; Hubbard *et al*, 2000c). There have been conflicting reports about the type of lung cancer seen in ILD patients and whether it differs from that seen in the general population. Some authors have reported no difference in the distribution of histological lung cancer types between patients with and without pulmonary fibrosis (Turner-Warwick *et al*, 1980; Park *et al*, 2001), while others have reported a distinct anatomical distribution of lung cancer in ILD patients, with most cancers in the peripheral lung (Matsushita *et al*, 1995; Ogura *et al*, 1997; Ma *et al*, 2001). Some authors have also reported a close relationship between the malignancy and honeycombed fibrotic areas (Matsushita *et al*, 1995; Hironaka and Fukayama, 1999).

### ILD in lung cancer patients

The more recent recognition that ILD develops in lung cancer patients after radiotherapy and chemotherapy (Takeuchi *et al*, 1996; Abid *et al*, 2001; Wang *et al*, 2001) has raised the question of whether the association is with the cancer or the treatment.

Therapy-associated ILD may help explain the higher prevalence of ILD in patient studies, as compared with population studies where often only palliative care and support were available. Furthermore, some reports of ILD in lung cancer patients may represent a worsening or an ‘exacerbation’ of a pre-existing chronic ILD that has been ‘triggered’ by the cancer therapy (Kawasaki *et al*, 2002; Yano *et al*, 2002).

In reports of hospital patient series from Korea and Japan, IPF was found in 2% of lung cancer patients (Kinoshita *et al*, 1990; Park *et al*, 2001) and in a review of several earlier Japanese studies, including that of Kinoshita *et al*, the overall frequency was approximately 3%, with some variation across studies (Takiguchi *et al*, 1993). Two other Japanese studies, an autopsy study and a retrospective study of patients following lung cancer resection, found IPF in 7–8% of these rather selected series of lung cancer cases (Hironaka and Fukayama, 1999; Kawasaki *et al*, 2002).

Lung cancer patients have an older age distribution than the general population and so would be expected to have a higher frequency of ILD. Diagnosis of ILD occurs mostly in the fifth and sixth decades, but differs between the ILD diseases, ranging from the fourth decade for sarcoidosis to the seventh decade for drug-related ILD (Lim *et al*, 1996; Johnston *et al*, 1997; Thomeer *et al*, 2001a, 2001b). Applying age-specific IPF rates from the Bernalillo County registry to a population of registered lung cancer cases in Sweden in 2000 (National Board of Health and Welfare, 2002), an expected IPF prevalence of around 0.07% and an expected IPF incidence of <0.04% per year were estimated. While these predicted rates in a population of lung cancer patients are over four times higher than in the general population, age distribution alone does not appear to explain the IPF frequencies of 2–8% in the selected lung cancer populations reported above.

### ILD as a complication of radiotherapy exemplifies the impact of past exposure to a risk factor

Radiation-associated ILD in non-small-cell lung cancer patients is well known and follows quite distinctive patterns. Of particular importance is the ability of radiation therapy to ‘condition’ the lung to develop ILD after an interval of time. Such a remote initiating factor can be discovered only if precise records of therapy are kept.

### Classical radiation pneumonitis following single-fraction radiotherapy

The histopathology of classical radiation pneumonitis after whole-lung radiotherapy with single doses of X-rays in mice and rats follows well-defined stages from the latent period, when damage is only visible by electron microscopy, through to the fibrosis phase, which develops from 6 months onwards (White, 1975). Classical radiation pneumonitis is an acute inflammatory response to lung radiotherapy and is confined to the treated area. Single-dose radiotherapies of the whole thorax in mice and half-body in humans have both shown a threshold dose for classical radiation pneumonitis and a narrow sigmoid dose–response curve with increasing morbidity and mortality over a very small dose range (Van Dyk *et al*, 1981; Travis and Tucker, 1986). In both animal and human studies, all subjects above the threshold dose eventually suffer irreversible pulmonary damage and death (Morgan and Breit, 1995).

### Sporadic radiation pneumonitis following fractionated radiotherapy

In contrast to classical radiation pneumonitis, clinical pneumonitis, which can occur after a fractionated course of radiotherapy to a portion of the lungs, is characterised by a nonproductive cough and dyspnoea disproportional to the volume of lung irradiated. The onset is unpredictable, occurs in only 5–10% of irradiated humans and usually resolves completely without long-term effects (Morgan and Breit, 1995). The term ‘sporadic radiation pneumonitis’ has been used to describe this clinical picture, which is distinct from classical radiation pneumonitis.

There is now evidence that sporadic pneumonitis is characterised by bilateral lymphocytic alveolitis and is actually a hypersensitivity pneumonitis similar to that seen in farmer's lung or methotrexate pneumonitis (Gibson *et al*, 1988; Abratt and Morgan, 2002).

**Figure 1:**
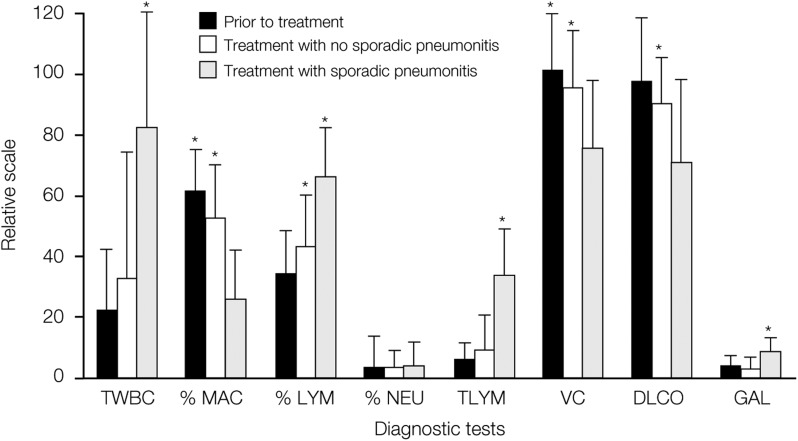
Changes in respiratory evaluation after unilateral thoracic radiotherapy, with and without sporadic radiation pneumonitis (reprinted from Morgan and Breit, 1995). Copyright © 1995 Elsevier. Relative values relate to numbers of cells per million per litre and absolute gallium lung scan values. ^*^Changes reaching statistical significance *P*<0.05. TWBC, total white blood cell count; MAC, macrophages; LYM, lymphocytes; NEU, neutrophilis; VC, vital capacity; DLCO, carbon monoxide transfer factor; GAL, gallium lung scan.

Following unilateral thoracic radiotherapy for breast cancer, bronchoalveolar lavage revealed increases in total cells and in lymphocytes in both the irradiated lung and the nonirradiated lung. Gallium scans also increased in intensity bilaterally (Gibson *et al*, 1988; Roberts *et al*, 1993). Although a lymphocytic alveolitis occurred in all patients, only a minority developed pneumonitis. It was noticed, however, that the increase in total lymphocytes and in the gallium index was more pronounced in women with pneumonitis. Retrospective analysis of the data revealed a more marked bilateral lymphocytic alveolitis and increase in gallium lung scan uptake in women with sporadic pneumonitis compared with those without pneumonitis (Figure 1

) (Morgan and Breit, 1995). The generalised lymphocytic response suggests an immunologically mediated response against damaged lung tissue, analogous to a hypersensitivity pneumonitis.

### Pulmonary fibrosis following radiotherapy

Pulmonary fibrosis is a consequence of the repair process following tissue injury by surgery, radiotherapy, chemotherapy, infection or acute and chronic inflammatory processes, although a precise mechanism is unknown.

Tissue repair involves recruitment of fibroblasts, cell replication and stimulation of fibroblasts to synthesise collagen. In inflammatory lung diseases, the spontaneous release of fibroblast growth factors, chemotactic factors, inflammatory cytokines and fibrogenic factors have all been documented. Irradiation of mononuclear phagocytes resulted in the dose-dependent synthesis and release of several growth factors for fibroblasts, including platelet-derived growth factor (PDGF) *β*, tumour necrosis factor *α* and insulin-like growth factor I. In addition, the *in vivo* irradiation of cells obtained by bronchoalveolar lavage from patients just prior to and 4–6 weeks after thoracic radiotherapy indicated a radiation-induced release of PDGF (Thornton *et al*, 1996). These results suggest that pulmonary fibrosis after therapeutic radiotherapy is not a consequence of direct damage from ionising radiation but, instead, arises from the local release of cytokines. It is therefore a separate event with a different aetiology from sporadic pneumonitis.

### ‘Exacerbations’ of ILD after radiation therapy

Asada and colleagues described two cases of ‘exacerbation’ of IP after radiation in patients with lung cancer and IP, diffusely outside the radiation field. Both patients died, and had also received chemotherapy (Asada *et al*, 1992).

### ILD as a complication of pulmonary resection

Some cases of ILD in lung cancer patients may occur as an exacerbation against a predisposing background of a pre-existing chronic IPF. For example, in one review of 50 patients with a diagnosis of both IIP and lung cancer who underwent pulmonary resection, 24% had an acute exacerbation within 30 days and the mortality in this group was very high (Yano *et al*, 2002). The rate of exacerbation was related to the extent of resection, as might be expected. However, although this was a small study, it is notable that no significant pre-operative predictive factors were found, although steroids were suggested to afford some protection. This review may be biased by the selection of patients reported in the literature. In another study of 711 consecutive lung cancer resections, 2 out of 53 (4%) with pre-existing IPF died post-operatively from exacerbation of IPF, corresponding to 2 out of 711 lung cancer patients (0.28%) (Kawasaki *et al*, 2002). In a third similar study of 2667 lung cancer thoracotomies, it was not reported what proportion had pre-existing IPF, but overall 8 (0.30%) died post-operatively from IP (Tanita *et al*, 2001), consistent with Kawasaki *et al*. Little is known about the rates of nonfatal ILD after operation. Although it is thus still unclear how common this complication is in lung cancer patients, and in particular in those with pre-existing IPF, it is possible that pre-existing ILD increases the risk of exacerbation after a lung trauma, due to specific disease processes or simply because of limited reserve capacity. Such pre-existing lung conditions might similarly modify susceptibility for other nonoperative treatments affecting the integrity of the lungs.

## SUMMARY

Standardised diagnostic methods and criteria that can be used in epidemiological studies are urgently needed to ensure consistency between studies. More prospective studies are required to understand the regional incidence and prevalence of ILD and the incidence and risk factors for lung cancer in ILD patients.

However, the picture is emerging of an increasing prevalence of ILD worldwide with some marked international differences. To explain this simply by improved diagnosis is not sufficient. There remain the important links between smoking and ILD, and the links between non-small-cell lung cancer and ILD. These raise the question of whether the treatment of lung cancer increases the risk of ILD, which also requires further research. But here there are important pointers to pre-existing ILD increasing the chances of an ‘exacerbation’, particularly with therapies such as radiation and thoracic surgery, but also potentially chemotherapy.
